# Demonstration of Thermally Tunable Multi-Band and Ultra-Broadband Metamaterial Absorbers Maintaining High Efficiency during Tuning Process

**DOI:** 10.3390/ma14195708

**Published:** 2021-09-30

**Authors:** Nanli Mou, Bing Tang, Jingzhou Li, Yaqiang Zhang, Hongxing Dong, Long Zhang

**Affiliations:** 1 Hangzhou Institute for Advanced Study, University of Chinese Academy of Sciences, Hangzhou 310024, China; mounanli@ucas.ac.cn (N.M.); lzhang@siom.ac.cn (L.Z.); 2 Key Laboratory of Materials for High-Power Laser, Shanghai Institute of Optics and Fine Mechanics, Chinese Academy of Sciences, Shanghai 201800, China; yaqiangzhang@siom.ac.cn; 3 Centre for Functional Photonics (CFP), Department of Materials Science and Engineering, City University of Hong Kong, Hong Kong 999077, China; tongsiom@mail.ustc.edu.cn; 4 CAS Center for Excellence in Ultra-Intense Laser Science, Shanghai 201800, China

**Keywords:** metamaterial absorber, high efficiency tunable absorber, vanadium dioxide, terahertz (THz), broadband absorber

## Abstract

Metamaterial absorbers (MMAs) with dynamic tuning features have attracted great attention recently, but most realizations to date have suffered from a decay in absorptivity as the working frequency shifts. Here, thermally tunable multi-band and ultra-broadband MMAs based on vanadium dioxide (VO_2_) are proposed, with nearly no reduction in absorption during the tuning process. Simulations demonstrated that the proposed design can be switched between two independently designable multi-band frequency ranges, with the absorptivity being maintained above 99.8%. Moreover, via designing multiple adjacent absorption spectra, an ultra-broadband switchable MMA that maintains high absorptivity during the tuning process is also demonstrated. Raising the ambient temperature from 298 K to 358 K, the broadband absorptive range shifts from 1.194–2.325 THz to 0.398–1.356 THz, while the absorptivity remains above 90%. This method has potential for THz communication, smart filtering, detecting, imaging, and so forth.

## 1. Introduction

Metamaterials are artificially-structured materials that possess a fantastic ability for controlling electromagnetic (EM) waves and can realize properties that are difficult with natural materials, such as negative refractive index, perfect lens, and invisible cloak [1,2,3]. In particular, metamaterial-based absorbers (MMA) have attracted great interest for their near perfect absorptivity, sub-wavelength scale, controllable working range, and great potential for wide range of applications, such as plasmonic sensors, solar cells, and thermal emitters [4,5,6,7]. A typical MMA, composed of metal/insulator/metal (MIM), was first proposed by Landy et al. [8], and which possessed dimension-dependent electric and magnetic responses. By designating the effective permittivity and permeability of the structure, the authors can tune the impedance of the MMA, matching free space, and thus eliminating reflection at the designed frequency. Due to the simplicity of the working mechanism and scalable features of metamaterials, a class of reflection-type MMAs consisting of an MIM sandwiched structure and ranging from microwaves, to THz, to the visible were soon proposed and demonstrated [7,9,10,11,12]. Generally the traditional resonance-based MPAs are narrowband and usually only have one absorption peak, which make the structures not applicable for many important applications, such as solar energy capture, electromagnetic stealth, and multi-spectra detection and imaging [13]. Due to the limitations of single-band absorbers, multi-band and broadband MMAs were later proposed, based on multiple resonators with different shapes or dimensions [14,15,16,17,18,19]. These absorbers possess fantastic properties, including high working efficiencies, ultra-thin thickness, and flat configurations, which are highly desirable in integrated-optics applications.

Despite the beneficial features of MMAs, most of the proposed designs so far have been based on materials with a fixed EM property. Once fabricated, these structures can only work with a fixed EM performance, which cannot meet the growing demand for smart devices. To make the properties of MMAs tunable, dynamic materials like graphene [20,21,22,23,24,25,26], liquid crystal [27,28,29], and phase change materials (PCMs) [30,31,32,33,34,35,36,37] have been introduced into the MMA structure. Via applying outside stimuli such as heat, electricity, and light the optical constant of these materials can be tuned, thus reshaping the EM performance of MMAs [37,38]. However, a big problem is that the shifting the frequency can lead to a mismatch in impedance, which limits the adjustable range with high efficiency. Additionally, in most cases, the real part (n) and imaginary part (k) of the refractive index both change during the process, which further enlarges the mismatch and leads to a sharp drop in absorptivity during the tuning process [39].

In this work, thermally tunable multi-band and ultra-broadband MMAs with nearly no decay in absorptivity during the tuning process are proposed, on the basis of vanadium dioxide (VO_2_). Compared with other active materials, VO_2_ features an insulator-to-metal transition (IMT) property, which exhibits an insulator state at room temperature and metallic state above the phase change temperature (~67 °C). Thanks to this special property, VO_2_ is widely used in many smart applications, such as tunable filters [40,41], thermal emitters [42,43,44], antennas [45], and multifunctional devices [46,47]. The basic design principle is the use of a 1-μm-thick VO_2_ film as a separate layer between two absorptive cavities. When the temperature-controlled VO_2_ phase state changes from insulator to metal, the lower and upper high-efficient MPA configurations can almost function independently. With properly designed geometric parameters, the proposed structure can work at room temperature and high temperature with different frequencies, with both having near-perfect absorption. Our simulations demonstrated that such a structure can achieve thermally tunable multiple perfect absorption, with the absorptivity remaining above 99.8%. By carefully designing the coupling of resonant modes, ultra-broadband switchable MMAs are demonstrated. At room temperature (298 K), the structure works with a >90% absorptivity, from 1.194–2.325 THz, and a relative absorptive bandwidth of (RAB) ~64.3%. When the temperature rises to 358 K, the >90% absorptivity work range shifts to 0.398–1.356 THz, and the RAB reaches ~109.2%, which is much broader than other reports. Our results can have potential applications in THz communication, detectors, smart filtering, and imaging, etc.

## 2. Model Construction and Simulation

**Figure 1:**
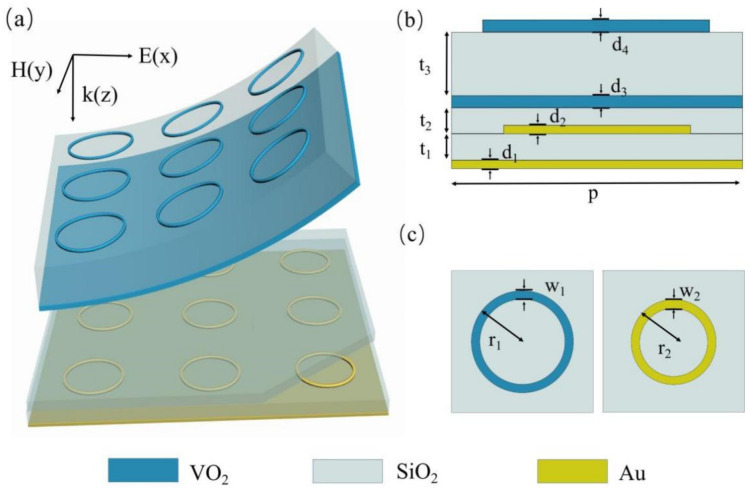
(**a**) Schematic of the proposed multi-band absorber. (**b**) Side view for one unit cell of the multi-layer composed structure. The detailed parameters are p = 85 µm, t_1_ = 6 µm, t_2_ = 5 µm, t_3_ = 20 µm, d_1_ = d_2_ = 0.4 µm, d_3_ = d_4_ = 1 µm. (**c**) Top view for one unit cell of periodic VO_2_ rings and Au rings, where r_1_ = 40 µm, w_1_ = 4 µm, r_2_ = 25 µm, w_2_ = 2 µm.

A schematic of the proposed multi-band absorber is shown in Figure 1, and the detailed parameters are listed in the caption. From bottom to top the structure consists of continuous Au film, an SiO_2_ dielectric layer, periodic Au rings, an SiO_2_ dielectric layer, continuous VO_2_ film, an SiO_2_ dielectric layer, and periodic VO_2_ rings. Due to the unique metal/insulator/metal (MIM) feature of VO_2_, the proposed metamaterial design can be safely divided into two independent working modes, according to the ambient temperature. At room temperature, VO_2_ exhibits transparent dielectric features, with the conductivity ~200 S/m; while the lower sandwich structure composed of periodic Au rings/SiO_2_ dielectric layer/continuous Au film contributes most to the interaction of incident EM. On the other hand, when the temperature rises to above 67 °C, the conductivity of VO_2_ increase to ~200,000 S/m and shows metallic features [48,49]. The 1 µm-thick metallic VO_2_ film is sufficient to eliminate transmission, and the EM features of the proposed absorber are decided by the upper sandwich structure, composed of periodic VO_2_ rings/SiO_2_ dielectric layer/continuous VO_2_ film. By designing the upper and lower sandwich structure, working as perfect absorbers under different frequencies, a thermally switchable perfect metamaterial absorber with no absorptivity decay can be achieved. Moreover, the independence of the two work modes makes the fabrication much easier and more flexible.

## 3. Results and Discussion

### 3.1. Absorption Characteristics of the Basic Unit Cell of the Proposed Thermally Switchable Absorber

We performed finite element method (FEM) numerical simulations using the commercial software COMSOL Multiphysics (version5.5) to investigate the proposed design. The dielectric permittivity of VO_2_ in the THz range can be described by the Drude model $\varepsilon \left(\omega \right)=\varepsilon_{\infty }-\omega_{p}^{2}\left(\sigma \right)/\left(\omega^{2}+i\gamma \omega \right)$ where $\varepsilon_{\infty }=12$ is the permittivity at high frequency, $\omega_{p}^{2}\left(\sigma \right)$ is the conductivity-dependent plasmon frequency, and $\gamma =5.75\times 10^{13}\;\mathrm{rad}/s$ stands for the collision frequency [33,50]. In addition, $\omega_{p}^{2}\left(\sigma \right)$ and σ are proportional to the free carrier density. At a specific conductivity $\sigma^{\prime }$, the plasmon frequency can approximately be defined by $\omega_{p}\left(\sigma^{\prime }\right)=\left(\sigma^{\prime }/\sigma_{0}\right)\omega_{p}^{2}\left(\sigma_{0}\right)$, where $\sigma_{0}=3\times 10^{5}\;S/m$, and $\omega_{p}\left(\sigma_{0}\right)=1.4\times 10^{15}\;\mathrm{rad}/s$, and as discussed in Section 2, at metallic and dielectric state $\sigma^{\prime }$ = 200,000 S/m and $\sigma^{\prime }$ = 200 S/m, respectively. SiO_2_ is modeled as a lossless dielectric material with permittivity ε=3.8. The conductivity of the gold is described by the Drude model, with the frequency $\omega_{p}=1.36\times 10^{16}\;\mathrm{rad}/s$, and the scattering rate $\Gamma =3.33\times 10^{13}$ [51].

**Figure 2:**
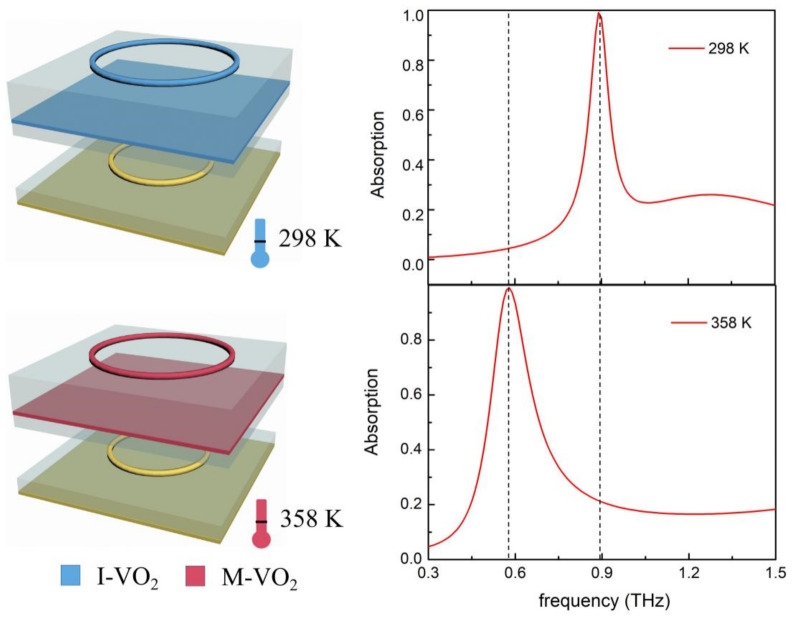
Absorption spectral characteristics of the thermally switchable absorber at 298 K and 358 K. I-VO_2_ represents the insulator state VO_2_, and M-VO_2_ represents the metallic state VO_2_.

Figure 2 shows the calculated absorption spectrum of the proposed device under 298 K (room temperature mode, corresponding to the dielectric state of VO_2_) and 358 K (high temperature mode, corresponding to the metallic state of VO_2_). At room temperature, the device exhibits ~99% perfect absorption at 0.89 THz. Since the dielectric VO_2_ has very limited interaction with incident THz EM, we consider that the perfect absorption spectrum is mainly decided by the lower Au-composed sandwich cavity. When the ambient temperature increases to 358 K, the VO_2_ transforms from a dielectric state to a metallic state, the upper VO_2_-composed sandwich cavity functions in trapping incident light, and the device showed a ~99% perfect absorption at 0.58 THz. This means that the thermally switchable absorption device can be switched between two frequencies with no decay in absorptivity. The physical mechanism of the perfect absorption with the two working modes can be explained by the impedance matched theory. The incident light is trapped by the metallic micro-structure and generates local surface plasmonic resonance (LSPR), which can be denoted by electric resonance. At the same time, the coupling effect between the metallic micro-structure and metallic film can generate magnetic resonance [8]. The metallic film can almost eliminate transmission, and by tuning the structural parameters, we can tune the impedance of the device to match the free space and prevent reflection; thus achieving perfect absorption [36]. The special aspect of our design is that we separated two thermally controllable and independent working modes, utilizing the IMT feature of VO_2_. The working frequency, efficiency, and even the fabrication process of the lower and upper sandwich structure can be independently configured, which makes the device much more practical.

**Figure 3:**
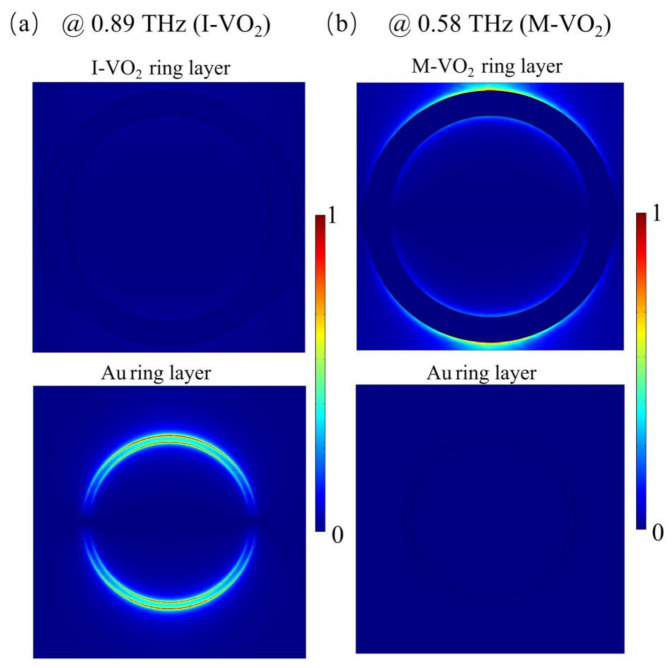
The electric field distributions of the VO_2_-ring array layer and Au-ring array layer at the absorption peaks when the VO_2_ is in the dielectric and metallic states.

In order to further investigate the underlying mechanism of the thermally switchable absorption spectrum, as shown in Figure 3, we calculated the electric field distribution at the absorption peaks in the VO_2_-ring array layer when the VO_2_ was in the insulator state (indicated by V layer) and the electric field distribution in the Au-ring array layer when the VO_2_ was in a dielectric state (indicated by A layer). Obviously, at room temperature (VO_2_ in dielectric state), the electric fields were mainly confined at the edge of the Au-rings, and the V layer showed no obvious field confinement. Similarly, at high temperature (VO_2_ in the dielectric state), the electric fields were mainly confined at the edge of the VO_2_-rings. The results show that the upper and lower sandwiched cavity structure can work independently via thermally switching the phase state of the VO_2_; thus, the two working modes can be independently designed and fabricated.

**Figure 4:**
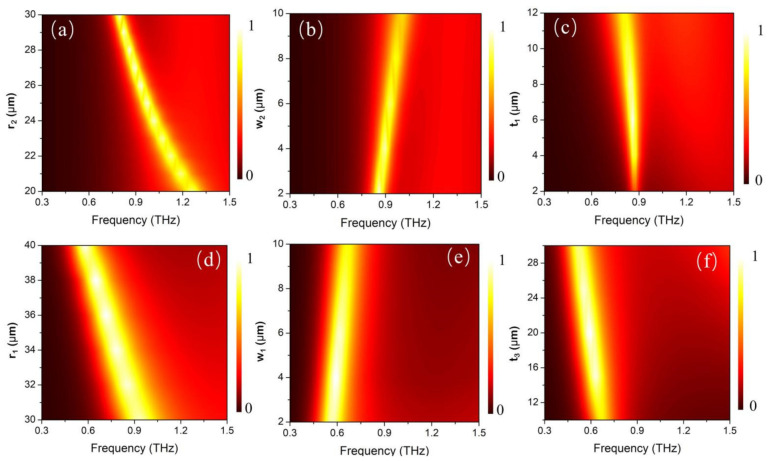
Absorption spectra of the composite structure as a function of the geometric parameters under room temperature (**a**–**c**), and at 358 K high temperature (**d**–**f**).

The functional performance of the upper and lower sandwiched cavity can also be tuned by changing the geometric parameters of the structure. In Figure 4, we calculated the absorption spectra of the composite structure as a function of the geometric parameters, under room temperature (Figure 4a–c) and at 358 K high temperature (Figure 4d–f). Figure 4a,d shows the absorption spectra as a function of the dimensions of metal/VO_2_ ring, when the VO_2_ is in the metal/dielectric state, respectively. When the diameter of the metal/VO_2_ ring increases, both working modes exhibit red shift and remain at >90% absorptivity. This is reasonable because the increase of ring diameter can lead to an increase of the effective distance of the resonant mode; thus, the resonant frequency exhibits red shift. Figure 4b,e shows the absorption spectra change with the width of the metal/VO_2_ ring under the two temperature modes. It can be seen that, under both modes, the absorption peaks exhibit blue shift with the increase of the ring width. Such a phenomenon can be explained by the hybridization plasmonic effect [52,53]. A ring dipole plasmonic mode can be analyzed as the hybridization of a disk and a hole dipole mode, resulting in a higher energy anti-bonding mode (M_1_) and a lower energy bonding mode (M_2_). When the width of the ring decreases, the coupling effect becomes stronger, which leads to a shift in frequencies of M_1_ and M_2_ to both sides; that is, M_1_ blue shifts and M_2_ red shifts. In this work, we were only concerned about the lower energy M_2_ mode, as with the increase of ring width, the coupling between disk dipole and hole dipole becomes weaker; thus, M_2_ exhibits a blue shift. Moreover, we noticed that with the variation of the ring width and diameter, the absorptivity will decrease or increase, resulting from the deviation from the optimized impedance matched condition. However, on the whole, several micron-scale fluctuations in dimensions have an insignificant influence on the absorptive performance, which makes the design robust for fabrication. The impedance matching condition for perfect absorption requires a permittivity of material to equal to the permeability, which greatly depends on the thickness of the dielectric layer. In Figure 4c,f, we calculated the absorption spectra as a function of the thickness of the dielectric layer t_1_ and t_3_. Similarly, the absorptivity increases at first and then decreases, further demonstrating the perfect absorption generated by the impedance matching mechanism. Through optimizing the dimensions of the MMAs, a near perfect absorption can be achieved, both in room temperature and higher temperature modes.

### 3.2. Thermally Switchable Multi-Band Absorber

**Figure 5:**
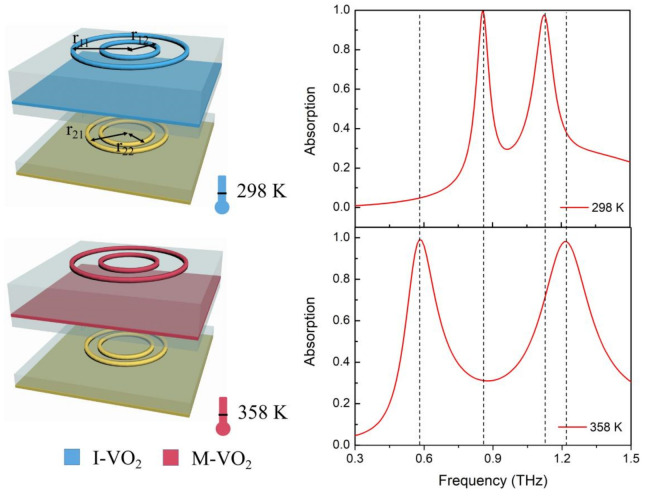
Schematic diagram and the absorption spectrum of the multi-band absorber at 298 K and 358 K. The parameters in the diagrams are r_11_ = 40 µm, r_12_ = 25 µm, r_21_ = 28 µm, r_22_ = 22 µm and the other geometric parameters of the structure are the same as those in Figure 2.

Based on the analysis of the basic thermally switchable perfect absorber, it is easy to expand the proposed design into a multi-band or even broadband switchable absorber by adding more Au/VO_2_ rings in the A layer and V layer. Figure 5 shows a schematic diagram and absorption spectrum of a multi-band absorber at 298 K and 358 K. Without changing the other parameters of the proposed basic structure, we added extra Au/VO_2_ rings in the A layer and V layer. A parameter sweep was carried out to optimize the design, and the geometric parameters of the rings are listed in the caption. Interestingly, as shown in Figure 5, we obtained two perfect absorption peaks, with >99.8% absorptivity, at 0.858 THz and 1.125 THz at room temperature. When the temperature rose to 358 K, the VO_2_ transformed to the metal state and the channel to the Au-based sandwich cavity was blocked; the VO_2_-based sandwich cavity functions to capture the incident light. Via tuning the geometric parameters of the resonant cavity, we obtained two perfect absorption peaks, with >99.8% absorptivity at 0.58 THz and 1.215 THz. Herein, through thermally switching the phase state of VO_2_, we obtained two switchable absorption peaks, which can maintain near-perfect absorptivity during the tuning process. Moreover, the number of perfect absorption peaks can be further improved by cascading more Au/VO_2_ rings, and the independence of the two working modes makes it possible for the device to function at two arbitrarily different frequency ranges, which endows the proposed absorber with potential for a wide range of applications.

### 3.3. Dynamically Tunable Ultra-Broadband Absorber with High Absorptivity

**Figure 6:**
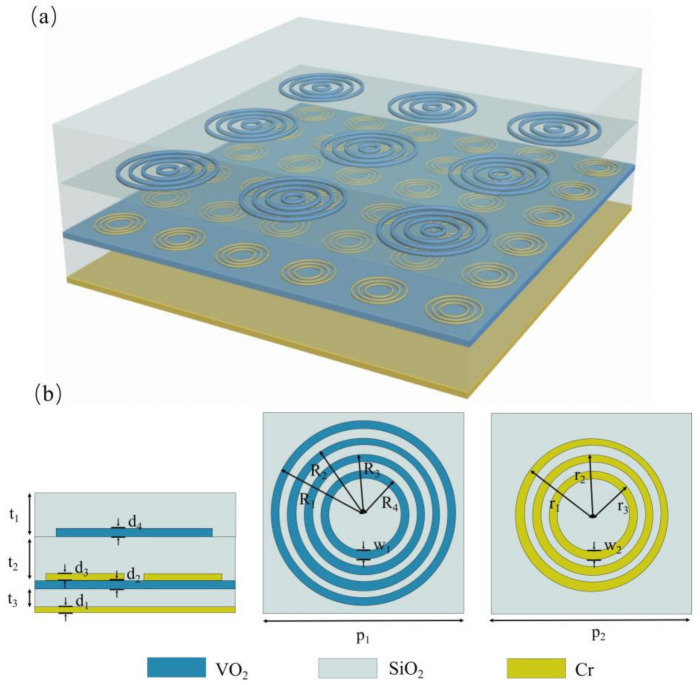
(**a**) Schematic of the ultra-broadband switchable absorber. (**b**) The unit cell of the metasurface composed of multiple layers. The dimensions are t_1_ = 13 µm, t_2_ = 25 µm, t_3_ = 39 µm, d_1_ = d_3_ = 0.3 µm, d_2_ = 1.1 µm, d_4_ = 0.5 µm, p_1_ = 90 µm, p_2_ = 45 µm, R_1_ = 42 µm, R_2_ = 34 µm, R_3_ = 25 µm, R_4_ = 19 µm, w_1_ = 2 µm, r_1_ = 15 µm, r_2_ = 11 µm, r_3_ = 8 µm, and w_2_ = 1 µm.

Most tunable broadband absorbers are based on a change of the refractive index of active materials, such as graphene and GST, under the stimulation of heat, electric, or light. The change in the real part of the refractive index mainly contributes to the frequency shift of the absorption peaks, while the variation of the imaginary part can result in a decay in absorptivity. In most cases both the real and imaginary parts are changed under external excitations, thus resulting in a decay of absorptivity. Via carefully designing the geometrical dimensions of multiple metal/VO_2_ rings and the thickness of the dielectric layer, an ultra-broadband switchable absorber without a reduction in absorptivity can be achieved. A schematic of the proposed design is shown in Figure 6. It is worth noting that, compared with the design in Figure 1, several modifications were made in order to achieve ultra-broadband absorption with high absorptivity, for both the upper and lower MPAs. First, we replaced the metal Au with high-loss Cr, for the relatively smaller Q factor of resonance. Second, the continuous VO_2_ film was placed below the Cr rings array, in order to further increase the loss and import a freely controllable parameter to optimize the broadband absorption property. Moreover, an extra layer of SiO_2_ was designed on top of the structure, which serves as an antireflection layer and helps to optimize the surface impedance. Broadband absorption can be achieved when the resonances of the different rings are close enough, thus four VO_2_ rings and three metal rings were designed in the upper and lower structure, respectively. Unlike the above discussed single or multiple narrowband absorption structures, which generally only concern the absorption peaks and can be safely designed independently for the upper and lower MPA working frequencies, for the broadband design some modifications needed to be carried out. The dielectric VO_2_ rings may slightly influence the surface impedance of the structure; thus, minor changes to the geometrical parameters were needed to optimize the surface impedance and flatten the top. The optimized dimensions of the rings are as follows: R_1_ = 42 µm, R_2_ = 34 µm, R_3_ = 25 µm, R_4_ = 19 µm, r_1_ = 15 µm, r_2_ = 11 µm, and r_3_ = 8 µm.

**Figure 7:**
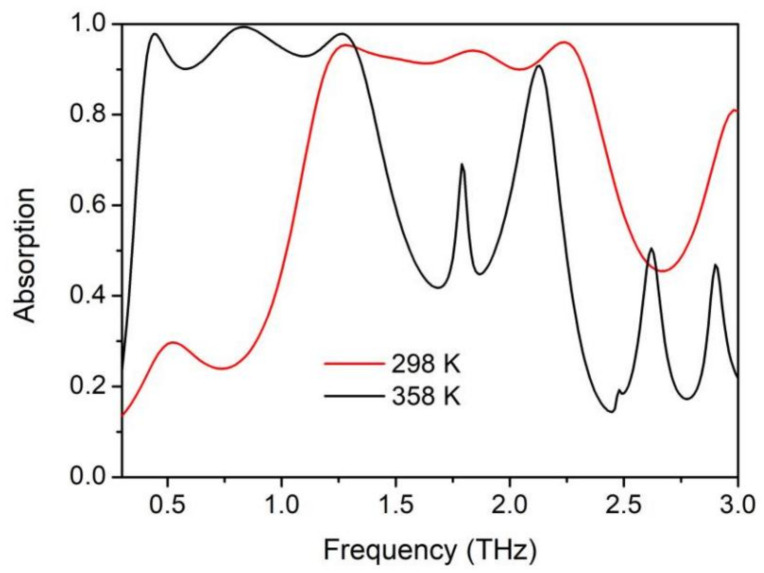
Absorption spectra at 298 K (conductivity of VO_2_ is about 200 S/m) and 358 K (conductivity of VO_2_ is about 200,000 S/m).

The absorption spectra of the proposed structure under room and high temperature are depicted in Figure 7. As discussed above, the VO_2_-film-seprated upper and lower cavity can work independently under different ambient temperatures. It is clear that, at room temperature, the structure exhibits broadband absorption from 1.194–2.325 THz with the absorptivity above 90%, and the relative absorptive bandwidth reaches (RAB) 64.3%. Under this condition, the VO_2_ film acts as a dielectric material, through which the incident wave can penetrate and enter into the lower structure, and the Cr-ring-based cavity works and interacts with the incident wave. When the temperature reaches 358 K, the VO_2_ transforms from a dielectric to a metal state. The THz EM wave can hardly penetrate the 1-µm-thick VO_2_ film, and the VO_2_-based cavity functions by capturing the incident light. Ultra-broadband absorptions from 0.398–1.356 THz, with absorptivity above 90%, are achieved, and the RAB reaches up to 109.2%. Compared with previous studies on tunable broadband absorbers, which inevitably suffered from a decay in absorptivity, our design can be thermally switched between two different broadband absorptive ranges, with the absorptivity remaining above 90%. By enlarging or reducing the dimensions to certain proportions, the working range of the upper and lower structures can be shifted to almost any target frequency.

## 4. Conclusions

In conclusion, thermally tunable polarization-insensitive multi-band and ultra-broadband MMAs, with nearly no decay in absorptivity during the tuning process are proposed based on VO_2_. Utilizing the IMT feature, we separated the structure into two independently designable temperature-dependent work modes, and each can function at different single (absorption peak shift from 0.58 THz to 0.89 THz) or multiple (absorption peaks shift from 0.858 THz and 1.125 THz to 0.58 THz and 1.215 THz) narrowband frequencies, with peak absorptivities above 99.8%. Compared to other tunable MMA designs, our structure can avoid the great decrease of absorptivity resulting from an impedance mismatch during the tuning process. Our calculations demonstrate that thermally switchable multiple absorption peaks with a absorptivity remaining above 99.8% can also be achieved. Furthermore, via introducing several cascading resonators with adjacent absorption spectra and carefully designing the coupling of the unit cells, ultra-broadband switchable perfect MMAs were also demonstrated. The proposed structure can be thermally shifted from 1.194–2.325 THz to 0.398–1.356 THz, with the absorptivity remaining above 90%, and the maximum RAB can reach 109.2%. Interestingly, such a design possesses great extensibility and flexibility. Owing to the independence of the upper and lower structures, the working range can be tuned further apart by scaling the dimensions; thus, arbitrary THz/THz or even infrared/THz multiplexing MMAs are achievable. It is worth mentioning that in this design we only focused on the final insulator and metal states of VO_2_; however, during the heating or cooling process, many continuous intermediate states also exist. Moreover, other special properties, such as thermal hysteresis, which may lead to a deviation of optical properties of the VO_2_ during the heating and cooling cycles [42], can also be utilized in the structure, to enrich the multifunctional applications. Benefiting from these attractive properties, the proposed structure may find many potential applications in smart filtering, THz imaging/detecting, and other multispectral devices.

## Data Availability

The data that support the findings of this study are available within.
